# Sleep During “Lockdown” in the COVID-19 Pandemic

**DOI:** 10.3390/ijerph17239094

**Published:** 2020-12-05

**Authors:** Athanasia Trakada, Pantelis T. Nikolaidis, Marilia dos Santos Andrade, Paulo José Puccinelli, Nicholas-Tiberio Economou, Paschalis Steiropoulos, Beat Knechtle, Georgia Trakada

**Affiliations:** 1 Department of Clinical Therapeutics, Division of Pulmonology, National and Kapodistrian University of Athens, School of Medicine, Alexandra Hospital, 11528 Athens, Greece; atrakada@gmail.com (A.T.); nt_economou@yahoo.it (N.-T.E.); gtrakada@hotmail.com (G.T.); 2 School of Health and Caring Sciences, University of West Attica, 12243 Athens, Greece; 3 Department of Physiology, Federal University of Sao Paulo, 05508-000 Sao Paulo, Brazil; marilia1707@gmail.com (M.d.S.A.); paulopuccinelli@hotmail.com (P.J.P.); 4 Department of Pulmonology, Democritus University of Thrace Medical School, University Hospital of Alexandroupolis, 68100 Alexandroupolis, Greece; steiropoulos@yahoo.com; 5 Institute of Primary Care, University of Zurich, 8091 Zurich, Switzerland; beat.knechtle@hispeed.ch

**Keywords:** Coronavirus Disease 2019 (COVID-19), lockdown, sleep, health professionals

## Abstract

The aim of this study was to determine if the lockdown measures applied due to the pandemic of Coronavirus Disease 2019 (COVID-19) affected the sleep of the general population and health professionals in six different countries (Greece, Switzerland, Austria, Germany, France, and Brazil). We used a web-based survey with a short questionnaire of 13 questions, translated into four languages (Greek, German, French, and Portuguese). The questionnaire included information about demographic and professional data, quantitative and qualitative characteristics of sleep, degree of abidance in lockdown measures, and data about illness or close contact with active confirmed cases of COVID-19. Initially, 2093 individuals participated. After exclusion of those who did not report their duration of sleep, the final sample comprised 1908 participants (Greek, *n* = 1271; German, *n* = 257, French, *n* = 48; Portuguese, *n* = 332), aged 42.6 ± 12.7 years, who were considered for further analysis. A main effect of the lockdown week on sleep duration was observed (+0.25 h; 95% confidence intervals, CI, 0.17, 0.32; *p* < 0.001), with the total sleep time of the lockdown week being longer than that under normal conditions. A week*occupation interaction on sleep duration was demonstrated (*p* < 0.001, η^2^ = 0.012). Sleep duration remained stable in health professionals (−0.18 h; 95% CI −0.36, 0.01; *p* = 0.063), whereas it increased in other occupations by 0.31 h (95% CI, 0.24, 0.39; *p* < 0.001). In terms of sleep quality, 15% of participants characterized their sleep as bad and 37.9% as average during the lockdown week. Almost 1 in 3 individuals (31.3%) reported worse quality of sleep during the lockdown week than under normal conditions. Sleep during the lockdown week was characterized as good by 47.1%, but only 38% of the health professionals group. In conclusion, the COVID−19 pandemic and lockdown affected sleep in different ways, depending on age, level of education, occupation, and country of residence.

## 1. Introduction

The first case of severe acute respiratory syndrome coronavirus 2 (SARS-CoV-2), which causes COVID-19, was reported in Wuhan, China on 31st of December 2019 [1,2]. The virus had spread rapidly, mostly within China, but also to other countries, including in the World Health Organization (WHO) European Region [3]. The first three detected European cases were reported in France on 24th of January 2020 and the first death was reported on 15th of February 2020, also in France [4]. As of the 21st of February, nine countries had reported cases: Belgium (1), Finland (1), France (12), Germany (16), Italy (3), Russia (2), Spain (2), Sweden (1), and the UK (9). The COVID-19 outbreak officially reached South America later. The coronavirus arrived in Brazil by plane by people from Europe and the first case was reported on February 26th and the first death was announced on March 17th in São Paulo state [5]. As new confirmed cases continued to increase worldwide, governments responded to COVID-19 using interventions that have imposed different degrees of restriction (“lockdown”) on the public, schools, and workplaces.

During an epidemic, people experience sudden and major changes in their daytime routines. They go through isolation, stress, insecurity about their health, worries about the situation and its duration, and anxiety regarding future job continuity and financial security [6]. Instead, limited working schedules may also mean that family members spend much more time together than usual or communicate more with friends through social networks. People can better organize their outdoor activities in order to be more exposed to daylight and get more exercise than normal. They can also develop sleep and work hours more closely aligned to their endogenous day and night rhythm, particularly evening types and adolescents [7,8].

This complicated situation is likely to affect sleep characteristics in terms of quantity and quality. Previous studies during this viral outbreak have not used specific sleep questionnaires and have mostly evaluated medical staff or those who have actually been exposed to or suffered from the virus itself, with a typical quarantine period of 14 days [9,10,11]. Our study aimed to evaluate the impact of lockdown due to the COVID-19 pandemic on sleep, in both the general population and health professionals, across several European countries (Greece, Switzerland, Austria, Germany, and France) and a South American country (Brazil).

## 2. Materials and Methods 

Our study was conducted between 25th of March and 6th of April 2020 in Greece, Switzerland, Austria, Germany, and France and between 10th and 14th June 2020 in Brazil, periods with similar restriction measures among these countries. The ethical committee of the “Alexandra” University Hospital, in Athens, Greece, approved the study protocol (Number of approval: 232/3 April 2020). We conducted a web-based, cross-sectional survey in order to prevent the spread of Severe Acute Respiratory Syndrome Coronavirus 2 (SARS-CoV-2) through droplets or contact. All people using social tools could see this survey and answer the questionnaire by clicking the relevant link [12]. 

Interested participants were interviewed by an ad hoc, simple, and anonymous questionnaire of our own development, which included 13 easy to answer questions, as follows: (1) Sex (male, female), (2) Age, (3) Place of residence, (4) Level of education (basic, upper secondary, higher-degree), (5) Current professional status (work outside home, limited work outside home, work at home, health professional, retirement), (6) Abidance in lockdown measures excluding work (yes, sometimes, no), (7) Mean hours of sleep per night during the last week (during lockdown), (8) Mean hours of sleep per night under normal conditions, (9) Qualitative characterization of sleep the last week (during lockdown) (good, moderate, bad), (10) Qualitative characterization of sleep the last week (during lockdown), when compared with sleep in normal conditions (same, better, worst), (11) Naps during the day in order to face the current situation (often, sometimes, never), (12) Need for hypnotic drugs or other substances in order to sleep (often, sometimes, never), and (13) illness or close contact with confirmed cases of COVID–19 (yes, no). The questionnaire was available in 4 languages—Greek, Portuguese, German, and French.

### 2.1. Participants

A total number of 2093 subjects completed the questionnaire. We excluded those who did not report duration of sleep during the lockdown week, resulting in a final sample of 1908 participants (Greek, *n* = 1271; German, *n* = 257, French, *n* = 48; Portuguese, *n* = 332), i.e., 1314 females (68.9%) and 594 males (31.1%), who were considered for further analysis. The mean age of the population was 42.6 ± 12.7 years old. Further, 286 subjects were health professionals (15.2%). Almost all were obedient in lockdown measures (91.7% yes, 6.1% sometimes), with only 2.2% of the participants reporting no obedience. COVID-19 infection or close contact with a confirmed case of COVID-19 was reported by 11.2% of the total population: i.e., 1.7% in Greece, 21.6% in German speaking countries: Switzerland/Austria/Germany, 38.8% in France, and 29.2% in Brazil. This was statistically more often reported by health professionals (23.1%) than the general population (*p* < 0.001).

### 2.2. Statistical Analysis

Statistical analyses were conducted using GraphPad Prism v. 7.0 (GraphPad Software, San Diego, CA, USA) and IBM SPSS v.23.0 (IBM SPSS Statistics for Windows, Version 23.0. Armonk, NY, USA: IBM Corp.). The statistical analysis included summarization of the data in tables and charts. Descriptive statistics procedures for complex survey data were used to examine demographic and sleep characteristics for all participants. The association among variables was examined by chi-square and the magnitude of these associations was tested by Cramér’s phi. A between-within analysis of variance examined the main effects of variables and their interaction on sleep duration and eta square tested the magnitude of these relationships. Pairwise t-tests and Cohen’s d also examined the effect of variables on sleep duration and their magnitude, respectively. A *p*-value less than 0.05 was regarded as statistically significant. 

## 3. Results

### 3.1. Sleep Quantity

**Figure 1:**
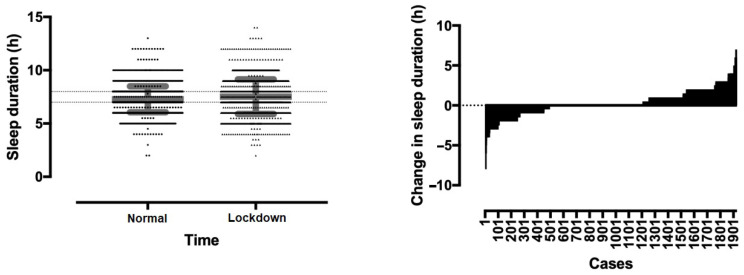
Distribution of sleep duration in normal and lockdown week (**left**) and change of sleep duration (**right**). On the left, the two parallel dashed lines show the range of 7–8 h sleep duration; error bars in grey show standard deviations; dots and horizontal lines show unique cases.

A main effect of lockdown week on sleep duration was observed (*p* < 0.001, η^2^ = 0.022) with the sleep duration of the lockdown week being longer than that under normal conditions (Figure 1). Sleep duration increased from 7.27 ± 1.21 h per day under normal conditions to 7.52 ± 1.62 h during the lockdown week (*p* < 0.001). The percentage of participants sleeping 7–8 h per day decreased from 66.1% previously to 48.6% in the lockdown week, whereas the percentage of those sleeping >8 h increased from 11.1% to 28.0%, respectively. The percentage of those sleeping <7 h was identical, 23.4% and 22.9%, respectively (Figure 1).

**Figure 2:**
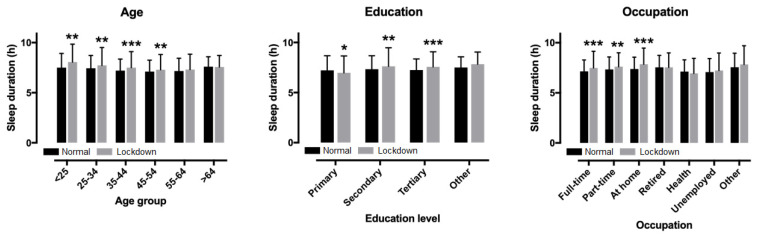
Changes in sleep duration from normal to the lockdown week by age, education, and occupation (* *p* < 0.05, ** *p* < 0.01, *** *p* < 0.001).

A main effect of education level on lockdown (*p* = 0.010, η^2^ = 0.007) and previous sleep duration (*p* < 0.001, η^2^ = 0.012) was also found, with the highest score in the primary education level. An education*time interaction on sleep duration was observed (*p* < 0.001, η^2^ = 0.013). The sleep duration increased in secondary education by 0.28 ± 1.82 h (95% CI 0.12, 0.45; *p* = 0.001) and in tertiary education by 0.32 ± 1.53 h (95% CI 0.23, 0.41; *p* < 0.001), while it decreased in primary education (−0.25 ± 1.71 h (95% CI −0.47, −0.03; *p* = 0.026) and did not change in other education (0.33 ± 1.39 h; 95% CI −0.04, 0.70; *p* = 0.081) (Figure 2).

### 3.2. Sleep Quality

Half of the population (47.1%) characterized their sleep during the lockdown week as good, 37.9% as average, and 15% as bad. In addition, one to two of the participants (49.5%) described the quality of sleep as the same, 19.2% as better, and 31.3% as worse than that under normal conditions. Need for hypnotic drugs or other substances in order to sleep was often 3.4%, occasionally 6.3%, and never 90.2%. Finally, taking naps during the day in order to face the current situation was often 11.9%, occasionally 36.7%, and never 51.4% of the total population. Data of the population are summarized in Table 1.

Participants who reported good quality of sleep during lockdown were older than those with average and bad quality of sleep (age 44.4 ± 12.7, 41.4 ± 12.5 and 40.3 ± 12.4 years, respectively; *p* < 0.001, η^2^ = 0.017). Education level was associated with both quality of sleep in lockdown (χ^2^ = 26.690, *p* < 0.001, φ = 0.118) and change in quality of sleep in lockdown compared to a normal week (χ^2^ = 20.146, *p* = 0.003, φ = 0.115) (Table 2). For instance, participants with tertiary level education had better quality of sleep in lockdown compared to those with a lower education level. In addition, participants with a tertiary level education reported better quality of sleep in lockdown compared to a normal week to a larger extent than those with a lower education level.

With regards to the relationship between quality and duration of sleep, participants with good quality of sleep in lockdown had a longer duration of sleep in lockdown (8.0 ± 1.3 h) than those with average (7.3 ± 1.5 h) and bad quality of sleep (6.4 ± 1.9 h; *p* < 0.001, η^2^ = 0.125). Change in sleep duration in lockdown compared to normal was 0.75 ± 1.27 h, 0.11 ± 1.55 h and −1.02 ± 2.07 h in participants with good, average, and bad quality of sleep in lockdown, respectively (*p* < 0.001, η^2^ = 0.137). However, these three groups of quality of sleep in lockdown did not differ in terms of sleep duration in a normal week (*p* = 0.106, η^2^ = 0.002).

### 3.3. Sleep and Age

A main effect of age group on lockdown sleep duration was observed (*p* < 0.001, η^2^ = 0.017), with the longest in the <25 years age group and the shortest in the 45–54 years group (Figure 2). A main effect of age group on previous sleep duration was also shown (*p* < 0.001, η^2^ = 0.016), with the longest in the >64 group and the shortest in the 45–54 group. No age group*time interaction on sleep duration was found (*p* = 0.078, η^2^ = 0.005), with the largest change in the <25 group (+0.55 h) and the smallest in the >64 group (−0.03 h). Age correlated with lockdown sleep duration (r = −0.093, *p* < 0.001), change in sleep duration (r = −0.063, *p* = 0.006), but not with previous sleep duration (r = −0.038, *p* = 0.095). 

### 3.4. Sleep in Men vs. Women

No sex difference was observed in sleep duration in the lockdown week (mean difference +0.14 h, 95% CI −0.01, 0.30; *p* = 0.075), whereas women had longer sleeps than men in previous weeks (+0.15 h, 95% CI 0.03, 0.26; *p* = 0.014). No sex*time interaction on sleep duration was shown (*p* = 0.956, η^2^ < 0.001). Sex was associated with compliance with self-restraint measures (χ^2^ = 6.218, *p* = 0.045, φ = 0.062), group of lockdown sleep duration (χ^2^ = 29.839, *p* < 0.001, φ = 0.136), group of previous sleep duration (χ^2^ = 11.180, *p* = 0.004, φ = 0.084), quality of sleep (χ^2^ = 42.325, *p* < 0.001, φ = 0.162), change of sleep quality (χ^2^ = 47.767, *p* < 0.001, φ = 0.175), naps (χ^2^ = 7.856, *p* = 0.020, φ = 0.070), and medication (χ^2^ = 11.697, *p* = 0.003, φ = 0.085). No sex association was shown with the occurrence of a COVID event in peers (χ^2^ = 0.083, *p* = 0.773, φ = −0.007). Compared to men, more women reported compliance with self-restraint measures, had less sleep duration of 7–8 h, bad sleep, decreased quality of sleep for the lockdown week when compared with the previous one, less naps, and higher use of medication.

### 3.5. Sleep and Occupation—General Population vs. Health Professionals 

**Figure 3:**
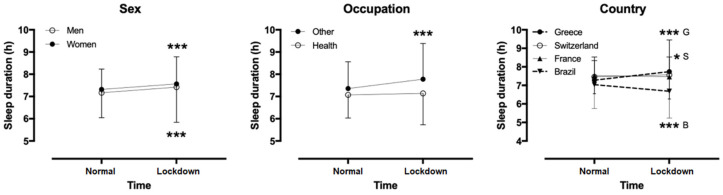
Changes of sleep duration from normal to lockdown week by sex, occupation, and country (* *p* < 0.05, *** *p* < 0.001; G = Greece, S = Switzerland/Austria/Germany, B = Brazil).

Considering two occupation groups (health professionals versus others), a week*occupation interaction on sleep duration was shown (*p* < 0.001, η^2^ = 0.012) (Figure 3). A pair-wise t-test showed that sleep duration increased in the lockdown week in other occupations by 0.31 ± 1.62 h (95% confidence intervals, CI, 0.24, 0.39; *p* < 0.001), whereas it remained stable in health professionals (−0.18 ± 1.60 h; 95% CI −0.36, 0.01; *p* = 0.063). Considering all occupation groups, a main effect of occupation on lockdown sleep duration was found (*p* < 0.001, η^2^ = 0.035), with the shortest in health professionals and the longest in unemployed people. Also, a main effect of occupation on previous sleep duration was observed (*p* < 0.001, η^2^ = 0.022), with the shortest in health professionals (6.94 ± 1.50 h) and the longest in retired people (7.84 ± 1.87 h) and those working at home (7.86 ± 1.60 h). A week*occupation interaction on sleep duration was shown (*p* = 0.012, η^2^ = 0.017), with the largest increase in those working at home (+0.48 ± 1.68 h) and the smallest in those working part-time out of home (+0.34 ± 1.62 h).

Occupation was associated with group of lockdown sleep duration (χ^2^ = 35.597, *p* < 0.001, φ = 0.150), quality of sleep (χ^2^ = 15.594, *p* < 0.001, φ = 0.100), change of sleep quality (χ^2^ = 6.676, *p* = 0.035, φ = 0.066), and occurrence of a COVID event in peers (χ^2^ = 12.961, *p* < 0.001, φ = 0.091). No occupation association was shown with compliance with self-restraint measures (χ^2^ = 2.937, *p* = 0.230, φ = 0.043), group of previous sleep duration (χ^2^ = 5.531, *p* = 0.063, φ = 0.060), naps (χ^2^ = 2.585, *p* = 0.275, φ = 0.041), and medication (χ^2^ = 1.480, *p* = 0.477, φ = 0.031). Compared to other occupations, health professionals had less sleep duration of 7–8 h, less good quality sleep, decreased quality of sleep, and higher occurrence of COVID in peers.

### 3.6. Sleep in Different Countries

A country*time interaction on sleep duration (*p* < 0.001, η^2^ = 0.036) was observed, with Greece showing the largest increase. Sleep duration increased in Greece by 0.45 ± 1.75 h (95% CI 0.35, 0.54; *p* < 0.001) and Switzerland/Austria/Germany by 0.10 ± 0.73 h (95% CI 0.01, 0.19; *p* = 0.029), did not change in France (−0.05 ± 0.99 h; 95% CI −0.34, 0.24; *p* = 0.719), and decreased in Brazil (−0.36 ± 1.60 h; 95% CI −0.53, −0.19; *p* < 0.001). Country was also associated with compliance with self-restraint measures (χ^2^ = 96.662, *p* < 0.001, φ = 0.223), group of lockdown sleep duration (χ^2^ = 52.412, *p* < 0.001, φ = 0.180), group of previous sleep duration (χ^2^ = 18.854, *p* < 0.001, φ = 0.109), quality of sleep (χ^2^ = 71.840, *p* < 0.001, φ = 0.194), change of sleep quality (χ^2^ = 91.796, *p* < 0.001, φ = 0.243), naps (χ^2^ = 9.642, *p* = 0.047, φ = 0.078), and occurrence of a COVID event in peers (χ^2^ = 309.441, *p* < 0.001, φ = 0.405). No country association was shown with medication (χ^2^ = 0.914, *p* = 0.923, φ = 0.024). Compared to other countries, Greece had higher compliance with self-restraint measures, less lockdown sleep duration of 7–8 h, better quality of sleep, larger change in quality of sleep, more naps, and less occurrence of COVID in peers.

## 4. Discussion

According to our study, the COVID-19 pandemic and lockdown impacted on sleep in different ways depending on age, level of education, occupation, and country of residence. Regarding quality, half of the population described their sleep as bad or average and one third of the participants reported worse sleep during the lockdown week than in a normal one. However, self-reported sleep duration increased in the general population, but not in health professionals. Individuals who preserved a good quality of sleep also slept more when compared to those with average and bad quality of sleep during the lockdown week, but not in a normal week. To our knowledge, this is the first study that evaluated possible associations between lockdown due to the COVID-19 pandemic and sleep characteristics, both in the general population and in health professionals, in several European countries and in Brazil. 

The COVID-19 outbreak in December 2019 and relevant mass home confinement led to a unique and stressful situation for many all around the globe. Lockdown separates persons potentially exposed to an infectious agent (and thus, at risk for disease) by restricting access to the general community. It has periodically been used for centuries to control the spread of infectious diseases, like cholera or plague, with some success [13,14]. It has always been associated with fear, threat, misunderstanding, and economic difficulties. A previous study during severe acute respiratory syndrome (SARS) in the early 2000s showed that persons placed in quarantine expressed anxiety, depression, stress, and posttraumatic stress disorder [15]. In the current COVID-19 outbreak and lockdown, another study showed that medical staff had increased levels of anxiety, stress, and self-efficacy, which were dependent on sleep quality and social support [11]. 

Although after a good night of sleep, one’s brain and body feels refreshed and restored to normal function, no study has focused specifically on sleep in the general population during the COVID pandemic and lockdown. Work time, family time, and time for socializing, relaxing, and leisure are the primary activities reciprocally related to sleep time. According to our data, a sleep “gain” existed during the current situation in a part of our population, possibly due to less activities. Adequate sleep is a key indicator of cardio-metabolic health. We hypothesize that this sleep “gain” could—at least partially—explain the observed decline of acute cardiovascular events. On the other hand, one third of our participants reported worse sleep quality when compared to that of a normal week and half of the population described their sleep quality as bad or average during the pandemic. Poor sleep quality has a major long term impact on mental and physical health and strongly correlates with depression and anxiety [16]. With regards to the relationship between quality and duration of sleep, participants with good quality of sleep in lockdown also had a longer duration of sleep in lockdown than those with average or bad quality of sleep. However, these three groups of quality of sleep in lockdown did not differ in terms of sleep duration in a normal week.

Sleep time was shown to be longer in young adults <25 years and shorter in the 45–54 years group. Young adults usually extend sleep duration during weekends as a recovery of sleep restriction during weekdays [7]. Also, sleep time reaches its minimum at age 45–54 years, when work time reaches its maximum [7]. The largest increase was observed in those working at home and the smallest in health professionals. According to previous data, each additional hour of work costs 7 to 10 min of sleep [17] and people working more than 50 h/week—e.g., health professionals—are more likely to be short sleepers and less likely to be long sleepers compared to people who work <35 h/week [18]. Furthermore, medical staff can suffer from insomnia during pandemics because of worries about being infected, uncertainty regarding effective disease control, and work isolation [19]. 

Sleep duration also increased in people with secondary and tertiary education, but not in those with primary and other education, in accordance with previously published data [19]. Moreover, a higher education level was associated with better quality of sleep when compared to other levels of education. A lower level of education is usually associated with a lower socioeconomic status and increased fear and anxiety about job continuity and financial security [19].

Finally, the observation that sleep increased more in Greece than in other countries may reflect the different ways of living and socializing in different countries of Europe; e.g., outdoor activities with family and/or friends are more common when the weather is warm and the sun is shining. In contrast to what was observed in Greece, in Brazil, the amount of sleeping hours decreased. There are several differences between countries that may be associated with this difference in sleeping hours’ behavior. The lower socioeconomic status of the Brazilians may increase the fear about their economic situation, which can increase concern about the economic situation compromising quality and quantity of sleep. Moreover, the Brazilian Ministry of Health recommended quarantine measures to avoid the coronavirus spread for the whole country, however a lot of people (including the Brazilian President, Jair Bolsonaro) denied the evidence about the severity of the disease [5,20]. The opposing recommendations from the Brazilian Ministry of Health and the country’s President increased the population’s feeling of insecurity, fear, and anxiety, which may also compromise the population’s sleep. In addition, Brazil has one of the highest transmission rates of the virus in the world [21] and together with EUA, one of the highest number of cases and deaths around the world [22], a fact that can increase the population’s concern about their health and be harmful for their sleeping hours.

No difference in absolute sleep length was observed between men and women before and during the “lockdown” week. However, fewer women than men slept 7–8 h, whereas more women than men reported bad sleep, decreased quality of sleep the last week when compared with the previous one, less naps, and higher use of medication. It is well documented that sleep in women differs in many aspects from that of men, mainly attributed to hormonal factors [23]. During their lifespan, women pass through different hormonal statuses—menstrual cycle, pregnancy, and menopause—with unique features of sleep disruption. In general, women appear to report a greater need for sleep and more subjective complaints of non-refreshing or insufficient sleep than men [23]. Also, insomnia is approximately 1.5 times more common in women than in men [24].

Our study has several limitations. First, our survey was based on a rapid, self-reported questionnaire to assess sleep characteristics. Subjective reports could not be verified with objective measurements, e.g., with actigraphy, due to quarantine and relevant mass home confinement. Also, the questionnaire was self-designed and not previously validated. No standard questionnaire exists for the investigation of sleep characteristics during a contagion outbreak. Finally, our population was not very well stratified. Participants were volunteers who responded through a web-based questionnaire and their distribution by country was not even. The main advantage of our study is the originality and novelty of our data. This is the first study to focus on the sleep quality of the general population during the COVID-19 outbreak.

## 5. Conclusions

According to our data, sleep was seriously affected during the COVID-19 pandemic and lockdown. A large percentage of the study population reported poor quality of sleep and less duration during the lockdown week when compared to a normal week. However, individuals who reported good quality of sleep in the lockdown week also slept more in lockdown than those with average or bad quality of sleep, but not health professionals. The related factors included age, level of education, occupation, and country of residence.

## Figures and Tables

**Table 1:** Demographic and sleep data of the study population (*n* = 1908).

Age (Years)	42.6 ± 12.7	*n*, %	
Sex (Female vs. Male)		1314 vs. 594	68.9 vs. 31.1
Compliance with self-restraint measures	Yes	1743	91.7
	Sometimes	116	6.1
	No	41	2.2
	Total	1900	100.0
Duration of sleep during lockdown week (hours)	7.52 ± 1.62		
Duration of sleep during a normal week (hours)	7.27 ± 1.21		
Quality of sleep during lockdown week	Good	898	47.1
	Average	723	37.9
	Bad	286	15.0
	Total	1907	100.0
Quality of sleep when compared to a normal week	Same	756	49.5
	Better	293	19.2
	Worst	477	31.3
	Total	1526	100.0
Napping in order to face current situation	Often	223	11.7
	Occasionally	685	36.0
	Never	993	52.2
	Total	1901	100.0
Medication or other substances in order to sleep	Often	93	4.9
	Occasionally	147	7.7
	Never	1666	87.4
	Total	1906	100.0
Illness or close contact to COVID 19	Yes	190	10.0
	No	1701	90.0
	Total	1891	100.0

**Table 2:** Quality of sleep and change in quality of sleep from normal to lockdown by education level.

Variable		Education level (*n* (%))			
		Primary	Secondary	Tertiary	Other
Quality of sleep in lockdown	Good	86 (37.4)	206 (43.8)	569 (49.7)	36 (64.3)
	Average	96 (41.7)	179 (38.1)	429 (37.5)	14 (25.0)
	Bad	48 (20.9)	85 (18.1)	147 (12.8)	6 (10.7)
	Total	230 (100.0)	470 (100.0)	1145 (100.0)	56 (100.0)
Quality of sleep in lockdown compared to a normal week	Same	27 (57.4)	204 (53.0)	482 (46.7)	40 (72.7)
	Better	5 (10.6)	65 (16.9)	216 (20.9)	6 (10.9)
	Worst	15 (31.9)	116 (30.1)	335 (32.4)	9 (16.4)
	Total	47 (100.0)	385 (100.0)	1033 (100.0)	55 (100.0)
